# A New Analytical Method for Solving the Fractional Swift-Hohenberg Equation with Atangana-Baleanu Derivative

**DOI:** 10.12688/f1000research.172948.1

**Published:** 2025-12-16

**Authors:** Jaafar Khalid Kareem, Hassan Kamil Jassim, Mohammed Taimah Yasser

**Affiliations:** 1 Department of Mathematics, University of Thi Qar, Thi Qar, +964, Iraq; 2 College of Technical Engineering, National University of Science and Technology, Thi-Qar, Iraq; 3 Department of Mathematics, Al-Ayen Iraqi University, Thi Qar, +964, Iraq

**Keywords:** Yasser-Jassim transform, Variational Itration Method, Swift-Hohnberg Equation, Atangana-Baleanu fractional derivative.

## Abstract

This work introduces a hybrid analytical technique for solving the Swift–Hohenberg equation by integrating the newly formulated Yasser–Jassim integral transform with the variational iteration method. The proposed framework is designed to efficiently handle both the classical and fractional forms of the equation, providing fast-convergent and highly accurate approximate solutions. To capture memory effects and nonlocal features more realistically, the Atangana–Baleanu fractional derivative in the Caputo sense is incorporated into the model. A rigorous convergence analysis is conducted, establishing sufficient conditions to ensure the stability, reliability, and accuracy of the iterative solutions. The performance of the method is assessed through a setiries of numerical experiments supported by graphical illustrations and tables of absolute error values, which collectively confirm the method’s superior accuracy and rapid convergence when compared with standard analytical approaches. Additionally, a detailed stability study of the fractional solutions is carried out and clearly verified through analytical arguments and numerical simulations. The results demonstrate that the combined Yasser–Jassim transform and variational iteration method offer a versatile and powerful tool for solving fractional-order partial differential equations. Beyond the Swift–Hohenberg equation, the proposed approach can be extended to a wide range of mathematical models, including nonlinear ordinary differential equations and integro-differential systems. Overall, the findings highlight the potential of this hybrid scheme to advance analytical methodologies within fractional calculus and nonlinear dynamical systems.

## 1. Introduction

The importance of fractional calculus (FC) is increasing due to its power in describing phenomena across various sciences such as physics, medicine, biology, and even engineering.

The power of fractional calculus lies in its ability to describe phenomena that require memory; that is to say, it describes phenomena that are influenced by past events or changes and their impact on the present or the immediate moment. This is a fundamental point in this field and its applications. ^1, 2, 19– 25^

A common example of this memory effect is cancer cells, where the mass and presence of cells depend not only on the current time but also on their past history of spread. Such a case cannot be accurately described by ordinary derivatives. ^1– 4^ The present work utilizes the YJDM method in order to construct series representations of solutions for 1D fractional variants of the Swift-Hohenberg equation, starting from its standard classical formulation.

$$\;\varpi_{t}=\gamma \varpi -{\left(1-\nabla^{2}\right)}^{2}\varpi +N\left(\varpi \right),\;x\;\in \;\mathbb{R},t>0$$ (1)

The Swift-Hohenberg equation, proposed in 1977 by J. Swift and P. Hohenberg, ^5^ serves as a general framework for modeling the evolution of velocity and temperature fields in thermal convection. In this formulation, $\overset{-}{\varpi }=\overset{-}{\varpi }\left(x,t\right)$ denotes a scalar field defined on either a plane or a line, γ represents the bifurcation parameter, and N(ϖ) stands for the nonlinear contribution. Beyond analyzing the influence of noise on bifurcations, defect dynamics, and spatiotemporal chaos, the Swift-Hohenberg equation has been widely employed to describe pattern formation in both simple systems, such as Rayleigh-Bénard convection, and in more intricate media, including biological tissues like the brain. ^6^ Its scope of application extends to various areas, including chemical and biological processes, hydrodynamics,laser physics, magneto-convection, and liquid-crystal light-valve experiments. ^7– 11^ Furthermore, the equation constitutes a fundamental model in understanding pattern formation in fluid layers constrained by horizontally conducting boundaries.

More precisely, we consider the following fractional versions of the S-H equation:

$${\;}^{C}D_{t}^{\theta }\varpi \left(x,t\right)+\frac{\partial^{4}\varpi \left(x,t\right)}{\partial x^{4}}+2\frac{\partial^{2}\varpi \left(x,t\right)}{\partial x^{2}}+\left(1-\alpha \right)\varpi \left(x,t\right)+\varpi^{3}\left(x,t\right)=0,$$ (2)

$${\;}^{C}D_{t}^{\theta }\varpi \left(x,t\right)+\frac{\partial^{4}\varpi \left(x,t\right)}{\partial x^{4}}-\kappa \frac{\partial^{3}\varpi \left(x,t\right)}{\partial x^{3}}+2\frac{\partial^{2}\varpi \left(x,t\right)}{\partial x^{2}}-\gamma \varpi \left(x,t\right)-2\varpi^{2}\left(x,t\right)+\varpi^{3}\left(x,t\right)=0,$$ (3)

and

$${\;}^{C}D_{t}^{\theta }\varpi \left(x,t\right)+\frac{\partial^{4}\varpi \left(x,t\right)}{\partial x^{4}}+2\frac{\partial^{2}\varpi \left(x,t\right)}{\partial x^{2}}+\left(1-\alpha \right)\varpi \left(x,t\right)-\varpi^{\ell }\left(x,t\right)+{\left(\frac{\partial \varpi \left(x,t\right)}{\partial x}\right)}^{\ell }=0$$ (4)

Here, ( x,t ) denotes the independent variables, α∈ℝ,ℓ≥0 , and 0<θ≤1 corresponds to the fractional order parameter, while κ and γ are interpreted as dispersion and bifurcation coefficients, respectively. Over the years, a variety of approximate solutions for different forms of the Swift-Hohenberg equation have been reported. For example, Akyildiz et al. ^12^ utilized the homotopy ana1ysis method (HAM) to establish solutions for its classical form (2). Khan et al. ^13^ applied both the differentia1 transform method (DTM) and the homotopy perturbation method (HPM) to study the time-fractional case of (2). In another contribution, the Adomian Laplace decomposition method (LADM) was utilized in ^14^ to investigate fractional generalizations (2)-(4). Similarly, Vishal and co-authors proposed approximate analytical treatments for nonlinear time-fractional S-H equations, whereas Merdan ^15^ applied the variational fractional iteration method(FVIM), incorporating the modified Riemann-Liouvi11e derivative, to obtain further approximate solutions. Moreover, a variety of sophisticated tools have been utilized to tackle both nolinear and linear (FPDEs). ^3, 16– 18, 27– 30^

In light of the increasing complexity introduced by fractional operators, the timefractional variant of the Swift-Hohnberg equation has attracted notable scholarly attention. Recent research trends have focused on constructing precise analytical frameworks capable of addressing the unique characteristics imposed by the fractional-order terms. These efforts have resulted in the development of several innovative techniques aimed at deriving meaningful and accurate solutions.

This study introduces a novel and efficient methodology for deriving analytical solutions to the fractional-order Swift-Hohenberg equation. The approach is computationally economical and methodologically straightforward, yielding reliable results with minimal analytical complexity. A convergence analysis validates the method, highlighting its practical utility. This work advances the development of solution techniques for fractional partial differential equations (FPDEs), which are critical to numerous scientific and engineering applications ^18, 33– 35^

Specifically, the study utilizes the recently developed Yasser-Jassim integral transform ^31^ in conjunction with the Variational Itration Method (VIM) to handle the fractional swiftHohnberg equation formulated with Atangana-Baleanu fractional derivatives. The procedure yields semi-analytical solutions represented in the form of rapidly converging series.

## 2. Preliminaries

Definition 2.1. The Mittag-Leffler function of two-parameter, denoted by $E_{\theta ,p}\left(z\right)$ , is a generalization of the exponential function and is given by the following series expansion ^34^:

Eθ,p(z)=∑n=0∞znΓ(nθ+p),θ,p,z∈ℂ,Re(θ)>0,Re(p)>0, (5)

Remark 2.1. Based on Definition 2.1, the following identities can be established:

$$\begin{array}{c} E_{2,1}\left(\kappa^{2}\right)=\cosh\left(\kappa \right)\\ E_{2,2}\left(\kappa^{2}\right)=\frac{\sinh\left(\kappa \right)}{\kappa }\\ E_{2,3}\left(\kappa^{2}\right)=\frac{1}{\kappa^{2}}\left[-1+\cosh\left(\kappa \right)\right] \end{array}$$

Definition 2.2. For a function ϖ(x) defined on x>0 , the Caputo derivative of order θ is expressed (see ^32^) as

$${\;}^{c}D_{x}^{\theta }\varpi \left(x\right)=\left\{ \begin{array}{ll} \frac{1}{\Gamma \left(n-\theta \right)}\int_{0}^{x}\;\;{\left(x-t\right)}^{n-\theta -1}\varpi^{\left(n\right)}\left(t\right)\mathrm{dt}, & n-1<\theta \;\leq \;n,n\;\in \;\mathbb{N}\\ \frac{d^{n}}{dx^{n}}\varpi \left(x\right), & \theta =n,n\;\in \;\mathbb{N} \end{array}\right.$$ (6)

Definition 2.3. Following, ^33^ the Atangana-Baleanu derivative of order θ for a function ϖ(t) on [a,t] is defined by

$${\;}^{\mathrm{AB}}D_{t}^{\theta }\varpi \left(t\right)=\frac{M\left(\theta \right)}{1-\theta }\int_{a}^{t}\;\;E_{\theta }\left(-\frac{\theta {\left(t-x\right)}^{\theta }}{1-\theta }\right)\varpi^{\prime }\left(x\right)\mathrm{dx},0<\theta <1$$ (7)

where the normalization function M(θ) obeys M(0)=M(1)=1 .

Definition 2.4. The Atangana-Baleanu integral of order θ is given in ^26, 33^ by

$${\;}^{\mathrm{AB}}I_{t}^{\theta }\varpi \left(t\right)=\frac{1-\theta }{M\left(\theta \right)}\varpi \left(t\right)+\frac{\theta }{M\left(\theta \right)\Gamma \left(\theta \right)}\int_{a}^{t}\;\;{\left(t-x\right)}^{\theta -1}\varpi \left(x\right)\mathrm{dx},\;0<\theta <1$$ (8)

With normalized function M(θ) .

Definition 2.5. (Yasser-Jassim Transform). The Yasser-Jassim transform of a function $\tilde{\varpi }\left(t\right)$ is introduced in ^31^ as:

$$H\left\{\varpi \left(t\right)\right\}=A\int_{0}^{\infty }\;\;e^{-\frac{1}{\sqrt{A}}t}\varpi \left(t\right)\mathrm{dt}$$ (9)

This transformation moves the function from the time domain to a new spectral domain determined by the parameter A .

**Some fundamental properties** ^31^:

1.
$H\left\{1\right\}=A\sqrt{A}\;$

2.
$H\left\{e^{\mathrm{bt}}\right\}=A\sqrt{A}\frac{1}{1-b\sqrt{A}}$ .
3.
$H\left\{E_{\theta }\left(\mathrm{bt}\right)\right\}=A\sqrt{A}\frac{1}{1-b{\sqrt{A}}^{\theta }}$ .
4.
$H\left\{t^{\theta }\right\}=A{\left(\sqrt{A}\right)}^{\theta +1}\Gamma \left(\theta +1\right).$

Lemma 2.1.

1. For the Caputo fractional derivative:
$$H\left\{{\;}^{c}D_{t}^{\theta }\varpi \left(t\right)\right\}=\frac{1}{{\left(\sqrt{A}\right)}^{\theta }}H\left\{\varpi \left(t\right)\right\}-\sum_{k=0}^{n-1}\;\;\frac{A}{{\left(\sqrt{A}\right)}^{\theta -k-1}}\varpi^{\left(k\right)}\left(0\right),n-1<\theta \;\leq \;n.$$ (10)
2. For the Atangana-Baleanu derivative:
$$H\left\{{\;}^{\mathrm{AB}}D_{t}^{\theta }\varpi \left(t\right)\right\}=\frac{M\left(\theta \right)}{1-\theta +\theta {\sqrt{A}}^{\theta }}\left[H\left\{\varpi \left(t\right)\right\}-A\sqrt{A}\varpi \left(0\right)\right]$$ (11)

Proof.

1. For the Caputo derivative:
$$H\left\{{\;}^{c}D_{t}^{\theta }\varpi \left(t\right)\right\}=\frac{1}{\Gamma \left(n-\theta \right)}H\left\{\int_{0}^{t}\;\;{\left(t-\tau \right)}^{n-\theta -1}\varpi^{\left(n\right)}\left(\tau \right)\mathrm{d\tau}\right\}$$

Applying the convolution property of the YJ transform ^31^:

$$=\frac{1}{A}H\left\{t^{n-\theta -1}\right\}\cdot H\left\{\varpi^{\left(n\right)}\left(t\right)\right\}$$

Substituting the transformation of the n th derivative yields:

$$=\frac{H\left\{\varpi \left(t\right)\right\}}{{\left(\sqrt{A}\right)}^{\theta }}-A\sum_{k=0}^{n-1}\;\frac{1}{{\left(\sqrt{A}\right)}^{\theta -k-1}}\varpi^{\left(k\right)}\left(0\right)$$

2. For the AB derivative:
$$H\left\{{\;}^{\mathrm{AB}}D_{t}^{\theta }\tilde{\varpi }\left(t\right)\right\}=H\left\{\frac{M\left(\theta \right)}{1-\theta }\int_{0}^{t}\;\;E_{\theta }\left(-\frac{\theta {\left(t-\tau \right)}^{\theta }}{1-\theta }\right)\varpi^{\prime }\left(\tau \right)\mathrm{d\tau}\right\}$$

Using the convolution theorem ^31^:

$$=\frac{M\left(\theta \right)}{1-\theta }\cdot \frac{1}{A}\cdot H\left\{E_{\theta }\left(-\frac{\theta t^{\theta }}{1-\theta }\right)\right\}\cdot H\left\{\varpi^{\prime }\left(t\right)\right\}$$

Upon simplification, we obtain:

$$=\frac{M\left(\theta \right)}{\theta {\sqrt{A}}^{\theta }+1-\theta }\left[H\left\{\varpi \left(t\right)\right\}-A\sqrt{A}\varpi \left(0\right)\right]$$

Theorem 2.1. (Banach’s Fixed Point Theorem) Let ( X,‖⋅‖ ) be a Hilbert space with X≠∅ . If T:X→X is a contraction on X , then T possesses a unique fixed point. ^14^

Theorem 2.2. Let (X,‖⋅‖) be a Hilbert space and T:X→X be a self-map satisfying ^14^

‖Tϰ−Tκ‖≤C‖ϰ−κ‖+β‖ϰ−κ‖,forallϰ,κ∈X,

where C≥0 and 0≤β<1 . Then, T is Picard T -stable.

## 3. Methodology of YJVIM

In this section, we will explain the algorithm of the new technique, Yasser-Jassim variational iteration Method (YJVIM). Consider the fractional S-H equation:

$${\;}^{\mathrm{AB}}D_{t}^{\theta }\varpi +\varpi_{\text{xxxx}}+2\varpi_{\mathrm{xx}}-\kappa \varpi_{\mathrm{xxx}}-\gamma \varpi +\left(1-\alpha \right)\varpi +N\left(\varpi \right)=0,\;0<\theta \;\leq \;1,$$ (12)

with:

ϖ(x,0)=g(x) (13)

We will use this formula in the paper because it allows us to express the three forms of the Swift-Hohenberg equation from it. For simplicity, we will denote the linear part by $M\left(\varpi \right)=\varpi_{\text{xxxx}}+2\varpi_{\mathrm{xx}}-\kappa \varpi_{\mathrm{xxx}}-\gamma \varpi +\left(1-\alpha \right)\varpi$ .

Now, by using the VIM ^22^ to Equation (12), we obtain:

$$\varpi_{n+1}=\varpi_{n}+\int_{0}^{t}\;\;\lambda \left(x,t-\zeta \right)\left[{\;}^{\mathrm{AB}}D_{t}^{\theta }\varpi_{n}+M\left(\varpi_{n}\right)+N\left(\varpi_{n}\right)\right]\mathrm{d\zeta}$$ (14)

Applying the YJ Transform

$$H\left\{\varpi_{n+1}\right\}=H\left\{\varpi_{n}\right\}+H\left\{\int_{0}^{t}\;\;\lambda \left(x,t-\zeta \right)\left[{\;}^{\mathrm{AB}}D_{t}^{\theta }\varpi_{n}+M\left(\varpi_{n}\right)+N\left(\varpi_{n}\right)\right]\mathrm{d\zeta}\right\}$$ (15)

Since the Lagrange multiplier depends on t−ζ and the equation written in terms of ζ , we apply the convolution theorem ^31^

$$H\left\{\varpi_{n+1}\right\}=H\left\{\varpi_{n}\right\}+\frac{1}{A}H\left\{\lambda \left(x,t\right)\right\}H\left\{{\;}^{\mathrm{AB}}D_{t}^{\theta }\varpi_{n}+M\left(\varpi_{n}\right)+N\left(\varpi_{n}\right)\right\},$$ (16)

Introducing $\frac{\delta }{\delta \omega_{n}}$ to both side of Equation (16):

$$\frac{\delta }{\delta \varpi_{n}}H\left\{\varpi_{n+1}\right\}=\frac{\delta }{\delta \varpi_{n}}H\left\{\varpi_{n}\right\}+\frac{1}{A}H\left\{\lambda \left(x,t\right)\right\}\frac{\delta }{\delta \varpi_{n}}H\left\{{\;}^{\mathrm{AB}}D_{t}^{\theta }\varpi_{n}+M\left(\varpi_{n}\right)+N\left(\varpi_{n}\right)\right\},$$ (17)

Considering the nonlinear terms as restricted variation $H\left\{\delta N\left(\varpi_{n}\right)\right\}=0$ , we have

$$H\left\{\delta \varpi_{n+1}\right\}=H\left\{\delta \varpi_{n}\right\}+\frac{1}{A}H\left\{\lambda \left(x,t\right)\right\}\frac{\delta }{\delta \varpi_{n}}H\left\{{\;}^{\mathrm{AB}}D_{t}^{\theta }\varpi_{n}\right\}$$ (18)

The optimally condition for $\varpi_{n+1}$ requires that $H\left\{\delta \varpi_{n+1}\right\}=0$ , leading that

$$0=H\left\{\delta \varpi_{n}\right\}+H\left\{\lambda \left(x,t\right)\right\}\frac{1}{A\left(1-\theta +\theta {\sqrt{A}}^{\theta }\right)}H\left\{\delta \varpi_{n}\right\}$$ (19)

then,

$$0=\left[1+H\left\{\lambda \left(x,t\right)\right\}\frac{1}{A\left(1-\theta +\theta {\sqrt{A}}^{\theta }\right)}\right]H\left\{\delta \varpi_{n}\right\}$$ (20)

and thus

$$H\left\{\lambda \left(x,t\right)\right\}=-A\left(1-\theta +\theta {\sqrt{A}}^{\theta }\right)$$ (21)

by substituting Equation (21) into Equation (16), we get

$$H\left\{\varpi_{n+1}\right\}=H\left\{\varpi_{n}\right\}-\left(1-\theta +\theta {\sqrt{A}}^{\theta }\right)H\left\{{\;}^{\mathrm{AB}}D_{t}^{\theta }\varpi_{n}+M\left(\varpi_{n}\right)+N\left(\varpi_{n}\right)\right\}$$ (22)

We apply the transformation to the equation and substitute in order to eliminate the fractional derivative,

$$H\left\{\varpi_{n+1}\right\}=A\sqrt{A}\varpi \left(x,0\right)-\left(1-\theta +\theta {\sqrt{A}}^{\theta }\right)H\left\{M\left(\varpi_{n}\right)+N\left(\varpi_{n}\right)\right\},$$ (23)

Finally, the application of the inverse YJ Transform yields the following recurrence relation:

$$\varpi_{n+1}=\varpi \left(x,0\right)-H^{-1}\left\{\left(1-\theta +\theta {\sqrt{A}}^{\theta }\right)H\left\{M\left(\varpi_{n}\right)+N\left(\varpi_{n}\right)\right\}\right\},$$ (24)

Accordingly, to obtain the solution of Equation (12), the Equation (24) yield an analytical solution in the form of an infinite series.

The solution can thus be expressed in the form:

$$\varpi \left(x,t\right)=\lim_{n\to \infty }\;\varpi_{n}$$ (25)

## 4. Stability analysis of YJVIM

Theorem 4.1. Let ( H,‖⋅‖ ) be a Hilbert space and T:H→H . Then, the iteration proceeds of YJVIM defined by:

$$T\left(\varpi_{n}\left(x,t\right)\right)=\varpi \left(x,0\right)-H^{-1}\left\{\left(1-\theta +\theta {\sqrt{A}}^{\theta }\right)H\left\{M\left(\varpi_{n}\right)+N\left(\varpi_{n}\right)\right\}\right\},n\;\geq \;1$$ (26)

is T -stab1e if $\beta =\left(\delta_{0}+\delta_{1}\right)\frac{\theta t^{\theta }}{\Gamma \left(\theta +1\right)}<1,\;0<\delta_{1},\delta_{0}<1.$

Proof. We begin by proving the existence of a fixed point for T . To this end, let n,m∈ℕ , and give thought to the two sequences of solutions defined as:

$$T\left(\varpi_{n}\left(x,t\right)\right)=\varpi \left(x,0\right)-H^{-1}\left\{\left(\theta {\sqrt{A}}^{\theta }-\theta +1\right)H\left\{M\left(\varpi_{n}\right)+N\left(\varpi_{n}\right)\right\}\right\}$$ (27)

$$T\left(\varpi_{m}\left(x,t\right)\right)=\varpi \left(x,0\right)-H^{-1}\left\{\left(\theta {\sqrt{A}}^{\theta }-\theta +1\right)H\left\{M\left(\varpi_{m}\right)+N\left(\varpi_{m}\right)\right\}\right\}$$ (28)

By subtracting (27) from (28), we obtain

$$T\left(\varpi_{n}\left(x,t\right)\right)-T\left(\varpi_{m}\left(x,t\right)\right)=H^{-1}\left\{\left(\theta {\sqrt{A}}^{\theta }-\theta +1\right)H\left\{M\left(\varpi_{m}\right)+N\left(\varpi_{m}\right)\right\}\right\}-H^{-1}\left\{\left(\theta {\sqrt{A}}^{\theta }-\theta +1\right)H\left\{M\left(\varpi_{n}\right)+N\left(\varpi_{n}\right)\right\}\right\},$$ (29)

Considering the norm on each side of (29), one may, without loss of generality, deduce that

‖T(ϖn(x,t))−T(ϖm(x,t))‖=‖H−1{(θAθ−θ+1)H{M(ϖm)+N(ϖm)}}−H−1{(θAθ−θ+1)H{M(ϖn)+N(ϖn)}}‖, (30)

Due to the linear nature of the YJ transform and its inverse, we arrive at:

$$\left\|T\left(\varpi_{n}\left(x,t\right)\right)-T\left(\varpi_{m}\left(x,t\right)\right)\right\|=\left\|H^{-1}\{\left(\theta {\sqrt{A}}^{\theta }-\theta +1\right)H\left\{M\left(\varpi_{n}\right)-M\left(\varpi_{m}\right)\right\}+N\left(\varpi_{n}\right)-N\left(\varpi_{m}\right)\}\right\|.$$ (31)

Applying the fundamental norm properties to (31), the derivation proceeds

$$\left\|T\left(\varpi_{n}\left(x,t\right)\right)-T\left(\varpi_{m}\left(x,t\right)\right)\right\|\;\leq \;\left\|H^{-1}\left\{\left(\theta {\sqrt{A}}^{\theta }-\theta +1\right)H\left\{M\left(\varpi_{n}\right)-M\left(\varpi_{m}\right)\right\}\right\}\right\|+\left\|H^{-1}\left\{\left(\theta {\sqrt{A}}^{\theta }-\theta +1\right)H\left\{N\left(\varpi_{n}\right)-N\left(\varpi_{m}\right)\right\}\right\}\right\|,$$ (32)

Now, assuming that

$$\left\|M\left(\varpi_{n}\right)-M\left(\varpi_{m}\right)\right\|\;\leq \;\delta_{0}\left\|\varpi_{n}-\varpi_{m}\right\|,\;\left\|N\left(\varpi \right)-N\left(\varpi \right)\right\|\;\leq \;\delta_{1}\left\|\varpi_{n}-\varpi_{m}\right\|,\;0<\delta_{1},\delta_{0}<1$$

Since 0<θ≤1 then, (32) becomes

$$\left\|T\left(\varpi_{n}\right)-T\left(\varpi_{m}\right)\right\|\;\leq \;\left(\delta_{0}\left\|\varpi_{n}-\varpi_{m}\right\|+\delta_{1}\left\|\varpi_{n}-\varpi_{m}\right\|\right)\left\|H^{-1}\left\{\left(\theta {\sqrt{A}}^{\theta }\right)H\left[1\right]\right\}\right\|$$ (33)

Observe that

$$H^{-1}\left\{\left(\theta {\sqrt{A}}^{\theta }\right)H\left[1\right]\right\}=\theta \frac{t^{\theta }}{\Gamma \left(\theta +1\right)}$$

Therefore, from (33), we have

$$\left\|T\left(\varpi_{n}\left(x,t\right)\right)-T\left(\varpi_{m}\left(x,t\right)\right)\right\|\;\leq \;\left(\delta_{0}+\delta_{1}\right)\theta \frac{t^{\theta }}{\Gamma \left(\theta +1\right)}\left\|\varpi_{n}-\varpi_{m}\right\|=\beta \left\|\varpi_{n}-\varpi_{m}\right\|,$$ (34)

Here, $\beta =\left(\delta_{0}+\delta_{1}\right)\theta \frac{t^{\theta }}{\Gamma \left(\theta +1\right)}<1$ . Therefore, the operator T admits a fixed point. Moreover, we verify that it fulfills the requirement stated in Theorem 2.2. In particular, we obtain

$$\left\|T\left(\varpi_{n}\left(x,t\right)\right)-T\left(\varpi_{m}\left(x,t\right)\right)\right\|\;\leq \;C\left\|\varpi_{n}\left(x,t\right)-\varpi_{m}(x,t)\right\|+\beta \left\|\varpi_{n}\left(x,t\right)-\varpi_{m}(x,t)\right\|,\;\text{for}\;C\;\geq \;0$$

This shows that the assumptions of the theorem are satisfied for the operator T . Therefore, according to Theorem 2.3, the YJVIM scheme is Picard T -stable whenever 0≤β<1 .

## 5. Convergence of YJ-VIN

In this part, we analyze the convergence of the newly proposed YJ-VIM method applied to the S-F equation discussed earlier. The necessary conditions ensuring the method’s convergence, as well as the associated error estimates, are outlined through the upcoming theorems.

Using Equation (31) to defined the following operator

$$A\left[\varpi \right]=\varpi \left(x,0\right)-\varpi_{n}-H^{-1}\left\{\left(1-\theta +\theta {\sqrt{A}}^{\theta }\right)H\left\{M\left(\varpi_{n}\right)+N\left(\varpi_{n}\right)\right\}\right\}$$ (35)

where $A\left[\bar{\varpi }\right]=\varpi_{n+1}-\varpi_{n}$ . For simplicity, let $M\left(\varpi \right)=\varpi_{\text{xxxx}}+2\varpi_{\mathrm{xx}}-\kappa \varpi_{\mathrm{xxx}}-\gamma \varpi +\left(1-\alpha \right)\varpi$ .

By simplifying and using the convolution property (see ^31^), we obtain the following equivalent formula:

$$A\left[\varpi \right]=\varpi \left(x,0\right)-\varpi_{n}-\left\{\left(1-\vartheta \right)\left[M\left(\varpi_{n}\right)+N\left(\varpi_{n}\right)\right]-\int_{0}^{t}\;\;\vartheta \frac{{\left(t-\tau \right)}^{\vartheta -1}}{\Gamma \left(\vartheta \right)}\left[M\left(\varpi_{n}\right)+N\left(\varpi_{n}\right)\right]\mathrm{d\tau}\right\}.$$

Defined the component ${℧}_{k},k=0,1,2,\ldots$ as

$$\begin{array}{c} {℧}_{0}=\varpi \left(x,0\right)\\ {℧}_{1}=A\left[{℧}_{0}\right]\\ \begin{array}{c} {℧}_{2}=A\left[{℧}_{0}+{℧}_{1}\right]\\ \vdots \\ {℧}_{k+1}=A\left[{℧}_{0}+{℧}_{1}+\ldots +{℧}_{k}\right] \end{array} \end{array}$$ (36)

Thus, it follows that $\;\varpi \left(x,t\right)=\lim_{n\to \infty }\;\varpi_{n}=\sum_{k=0}^{\infty }\;{℧}_{k}$ . Accordingly, the solution to the problem (12)-(13) can be expressed as

$$\varpi \left(x,t\right)=\sum_{k=0}^{\infty }\;\;{℧}_{k}$$ (37)

Where the initial approximation ${℧}_{0}=\varpi \left(x,0\right)$ .

Theorem 5.1. Let A be an operator such that $A:H^{1}\to H^{1}$ . The series solution $\varpi \left(x,t\right)=\sum_{k=0}^{\infty }\;{℧}_{k}$ converges provided that there exists a constant 0<γ<1 satisfying $\left|{℧}_{k+1}\right|\;\leq \;\gamma \left|{℧}_{k}\right|$ for all k=0,1,2,… , where $H^{1}$ denotes a Hilbert space.

Proof. Let define the sequence ${\left\{S_{n}\right\}}_{n=0}^{\infty }$ as

$$\begin{array}{c} G_{0}={℧}_{0}\\ G_{1}={℧}_{0}+{℧}_{1}\\ \begin{array}{c} G_{2}={℧}_{0}+{℧}_{1}+{℧}_{2}\\ \vdots \\ G_{n}={℧}_{0}+{℧}_{1}+\ldots +{℧}_{n} \end{array} \end{array}$$ (38)

In order to prove that the sequence ${\left\{G_{n}\right\}}_{n=0}^{\infty }$ forms a Cauchy sequence in the Hilbert space $H^{1}$ , we consider

$$\left\|G_{n+1}-G_{n}\right\|=\left\|{℧}_{n+1}\right\|\;\leq \;\gamma \left\|{℧}_{n}\right\|\;\leq \;\gamma^{2}\left\|{℧}_{n-1}\right\|\ldots \;\leq \;\gamma^{n+1}\left\|{℧}_{0}\right\|$$ (39)

For every n,j∈N,n≥j we have

$$\begin{array}{c} \left\|G_{n}-G_{j}\right\|=\left\|G_{n}-G_{n-1}+G_{n-1}-G_{n-2}+\ldots +S_{j+1}-S_{j}\right\|\;\\ \;\leq \;\left\|G_{n}-G_{n-1}\right\|+\left\|G_{n-1}-G_{n-2}\right\|+\ldots +\left\|S_{j+1}-S_{j}\right\|\\ \begin{array}{c} \;\leq \;\gamma^{n}\left\|{℧}_{0}\right\|+\gamma^{n-1}\left\|{℧}_{0}\right\|+\ldots +\gamma^{j+1}\left\|{℧}_{0}\right\|\;\\ =\frac{1-\gamma^{n-j}}{1-\gamma }\gamma^{j+1}\left\|{℧}_{0}\right\| \end{array} \end{array}$$ (40)

Because 0<γ<1 , it follows that $\lim_{n,j\to \infty }\;\left\|G_{n}-G_{j}\right\|=0$ . Therefore, the sequence ${\left\{G_{n}\right\}}_{n=0}^{\infty }$ is Cauchy in the Hilbert space, ensuring the convergence of the series solution $\overset{-}{\varpi }\left(x,t\right)=\sum_{k=0}^{\infty }\;{℧}_{k}$ .

Theorem 5.2. If the series $\overset{-}{\varpi }\left(x,t\right)=\sum_{k=0}^{\infty }\;{℧}_{k}$ converges, then it represents the exact solution to the problem (12)-(13)

Proof. Assuming the series solution converges, denoted by $\phi \left(x,t\right)=\sum_{k=0}^{\infty }\;{℧}_{k}$ , it follows that, $\lim_{j\to \infty }\;{℧}_{j}=0,\sum_{j=0}^{n}\;\left[{℧}_{j+1}-{℧}_{j}\right]={℧}_{n+1}-{℧}_{0}$ and so,

$$\sum_{j=0}^{\infty }\;\;\left[{℧}_{j+1}-{℧}_{j}\right]=\lim_{j\to \infty }\;{℧}_{j}-{℧}_{0}=-{℧}_{0}$$ (41)

Utilizing the operator ${\;}^{\mathrm{AB}}D_{t}^{\vartheta }$

$${\sum_{j=0}^{\infty }}^{\mathrm{AB}}D_{t}^{\vartheta }\left[{℧}_{j+1}-{℧}_{j}\right]=0$$ (42)

Meanwhile, based on definition (36), we obtain

$${}^{\mathrm{AB}}D_{t}^{\vartheta }\left[{℧}_{j+1}-{℧}_{j}\right]={\;}^{\mathrm{AB}}D_{t}^{\vartheta }\left[A\left[{℧}_{0}+{℧}_{1}+\ldots +{℧}_{j}\right]-A\left[{℧}_{0}+{℧}_{1}+\ldots +{℧}_{j-1}\right]\right]$$ (43)

provided that j≥1 it follows from definition (35) that

$${\;}^{\mathrm{AB}}D_{t}^{\vartheta }\left[{℧}_{j+1}-{℧}_{j}\right]={\;}^{\mathrm{AB}}D_{t}^{\vartheta }\left[-\left[{℧}_{j}\right]-\left(1-\vartheta \right)\{M\left({℧}_{0}+\ldots +{℧}_{j}\right)+N\left({℧}_{0}+\ldots +{℧}_{j}\right)-M\left({℧}_{0}+\ldots +{℧}_{j-1}\right)-N\left({℧}_{0}+\ldots +{℧}_{j-1}\right)\}-\int_{0}^{t}\;\;\vartheta \frac{{\left(t-\tau \right)}^{\vartheta -1}}{\Gamma \left(\vartheta \right)}\{M\left({℧}_{0}+\ldots +{℧}_{j}\right)+N\left({℧}_{0}+\ldots +{℧}_{j}\right)-M\left({℧}_{0}+\ldots +{℧}_{j-1}\right)-N\left({℧}_{0}+\ldots +{℧}_{j-1}\right)\}\mathrm{d\tau}\right],$$ (44)

We observe in Equation (44) that when applying the operator ${\;}^{\mathrm{AB}}D_{t}^{\vartheta }$ , the second and third terms are the inverse of the operator, resulting in the following:

$${\;}^{\mathrm{AB}}D_{t}^{\vartheta }\left[{℧}_{j+1}-{℧}_{j}\right]=-{\;}^{\mathrm{AB}}D_{t}^{\vartheta }\left[{℧}_{j}\right]-\{M\left({℧}_{0}+\ldots +{℧}_{j}\right)+N\left({℧}_{0}+{℧}_{1}+\ldots +{℧}_{j}\right)-M\left({℧}_{0}+{℧}_{1}+\ldots +{℧}_{j-1}\right)-N\left({℧}_{0}+{℧}_{1}+\ldots +{℧}_{j-1}\right)\},j\;\geq \;1$$

Consequently, we have

$$\begin{array}{c} \sum_{j=0}^{n}{\;}^{\mathrm{AB}}D_{t}^{\vartheta }\left[{℧}_{j+1}-{℧}_{j}\right]=-{\;}^{\mathrm{AB}}D_{t}^{\vartheta }\left[{℧}_{0}\right]-\left\{M\left({℧}_{0}\right)+N\left({℧}_{0}\right)\right\}\\ -{\;}^{\mathrm{AB}}D_{t}^{\vartheta }\left[{℧}_{1}\right]-\left\{M\left({℧}_{0}+{℧}_{1}\right)+N\left({℧}_{0}+{℧}_{1}\right)-M\left({℧}_{0}\right)-N\left({℧}_{0}\right)\right\}\\ \begin{array}{c} -{\;}^{\mathrm{AB}}D_{t}^{\vartheta }\left[{℧}_{2}\right]-\{M\left({℧}_{0}+{℧}_{1}+{℧}_{2}\right)+N\left({℧}_{0}+{℧}_{1}+{℧}_{2}\right)-M\left({℧}_{0}+{℧}_{1}\right)-N\left({℧}_{0}+{℧}_{1}\right)\}\\ \vdots \\ \begin{array}{c} -{\;}^{\mathrm{AB}}D_{t}^{\vartheta }\left[{℧}_{n}\right]-\{M\left({℧}_{0}+\ldots +{℧}_{n}\right)+N\left({℧}_{0}+\ldots +{℧}_{n}\right)\\ -M\left({℧}_{0}+\ldots +{℧}_{n-1}\right)-N\left({℧}_{0}+\ldots +{℧}_{n-1}\right)\} \end{array} \end{array} \end{array}$$

Therefore,

$$\sum_{j=0}^{\infty }{\;}^{\mathrm{AB}}D_{t}^{\vartheta }\left[{℧}_{j+1}-{℧}_{j}\right]=-{\;}^{\mathrm{AB}}D_{t}^{\vartheta }\left\{\sum_{j=0}^{\infty }\;\;{℧}_{j}\right\}-M\left\{\sum_{j=0}^{\infty }\;\;{℧}_{j}\right\}-N\left\{\sum_{j=0}^{\infty }\;\;{℧}_{j}\right\}$$ (45)

From Equations (42) and (45), observe that the solution series represents the exact solution to Problem (19)-(20).

Theorem 5.3. Assume the infinite series

$$\sum_{k=0}^{\infty }\;{℧}_{k}$$

converges to the exact solution ϖ(x,t) . If one uses the partial sum

$$\sum_{k=0}^{j}\;{℧}_{k}$$

as an approximation, then the truncation error $E_{j}\left(x,t\right)$ can be bounded by

$$E_{j}\left(x,t\right)\;\leq \;\frac{\gamma^{j+1}}{1-\gamma }\left\|{℧}_{0}\right\|$$

Proof. From inequality (40), we have

$$\left\|S_{n}-S_{j}\right\|\;\leq \;\frac{1-\gamma^{n-j}}{1-\gamma }\gamma^{j+1}\left\|{℧}_{0}\right\|,$$ (46)

For n≥j , Now, as n→∞ then $S_{n}\to \varpi \left(x,t\right)$ . So,

$$\left\|\varpi \left(x,t\right)-\sum_{k=0}^{j}\;\;{℧}_{k}\right\|\;\leq \;\frac{1-\gamma^{n-j}}{1-\gamma }\gamma^{j+1}\;\left\|{℧}_{0}\right\|,$$ (47)

Also, since 0<γ<1 we have $\left(1-\gamma^{n-j}\right)<1$ , Then, we conclude

$$\left\|\varpi \left(x,t\right)-\sum_{k=0}^{j}\;\;{℧}_{k}\right\|\;\leq \;\frac{1}{1-\gamma }\gamma^{j+1}\;\left\|{℧}_{0}\right\|.$$ (48)

## 6. Illustrative example

In this part of paper, we solve three numerical examples utilizing the Atangana-Baleanu fractional operator and present corresponding graphs and tables of the absolute error.

Example 6.1. Consider the linear fractional S-H equation:

$${\;}^{\mathrm{AB}}D_{t}^{\theta }\varpi +\left(1-\alpha \right)\varpi +2\varpi_{x}+\varpi_{\text{xxxx}}=0,\;0<\theta \;\leq \;1,$$ (49)

with the initial condition:

ϖ(x,0)=sinx (50)

Solution. Based on the above method derivation, Equation (24) provides the foundation for determining the iterations, the solution proceeds as follows:

$$\varpi_{0}=\sin\left(x\right)$$

$$\begin{array}{c} \varpi_{1}=\varpi \left(x,0\right)-H^{-1}\left[\left({\sqrt{A}}^{\theta }+1-\theta \right)H\left\{\left(1-\alpha \right)\varpi_{0}+2\varpi_{0_{x}}+\varpi_{0_{\text{xxxx}}}\right\}\right]\\ =\sin\left(x\right)+\alpha \sin\left(x\right)\left(\frac{\theta t^{\theta }}{\Gamma \left(\theta +1\right)}+1-\theta \right), \end{array}$$

$$\begin{array}{c} \varpi_{2}=\varpi \left(x,0\right)-H^{-1}\left[\left(\theta {\sqrt{A}}^{\theta }+1-\theta \right)H\left\{\left(1-\alpha \right)\varpi_{1}+2\varpi_{1_{x}}+\varpi_{1_{\text{xxxx}}}\right\}\right]\;\\ =\sin\left(x\right)\left[1+\alpha \left(\frac{\theta t^{\theta }}{\Gamma \left(\theta +1\right)}+1-\theta \right)+\alpha^{2}\left(\frac{\theta^{2}t^{2\theta }}{\Gamma \left(2\theta +1\right)}+2\left(1-\theta \right)\frac{\theta t^{\theta }}{\Gamma \left(\theta +1\right)}+{\left(1-\theta \right)}^{2}\right)\right], \end{array}$$

$$\begin{array}{c} \varpi_{3}=\sin\left(x\right)[1+\alpha \left(\frac{\theta t^{\theta }}{\Gamma \left(\theta +1\right)}+1-\theta \right)+\alpha^{2}\left(\frac{\theta^{2}t^{2\theta }}{\Gamma \left(2\theta +1\right)}+2\left(1-\theta \right)\frac{\theta t^{\theta }}{\Gamma \left(\theta +1\right)}+{\left(1-\theta \right)}^{2}\right)\\ +\alpha^{3}\left[{\left(1-\theta \right)}^{3}+3{\left(1-\theta \right)}^{2}\frac{\theta t^{\theta }}{\Gamma \left(\theta +1\right)}+3\left(1-\theta \right)\theta^{2}\frac{t^{2\theta }}{\Gamma \left(2\theta +1\right)}+\frac{\theta^{3}t^{3\theta }}{\Gamma \left(3\theta +1\right)}\right]], \end{array}\;$$

The solution can be written in series form:

$$\begin{array}{c} \varpi \left(x,t\right)=\sin\left(x\right)+\alpha \sin\left(x\right)\left(1-\theta +\frac{\theta t^{\theta }}{\Gamma \left(\theta +1\right)}\right)\\ +\alpha^{2}\sin\left(x\right)\left[{\left(1-\theta \right)}^{2}+2\left(1-\theta \right)\frac{\theta t^{\theta }}{\Gamma \left(\theta +1\right)}+\frac{\theta^{2}t^{2\theta }}{\Gamma \left(2\theta +1\right)}\right]\\ +\alpha^{3}\sin\left(x\right)\left[{\left(1-\theta \right)}^{3}+3{\left(1-\theta \right)}^{2}\frac{\theta t^{\theta }}{\Gamma \left(\theta +1\right)}+3\left(1-\theta \right)\theta^{2}\frac{t^{2\theta }}{\Gamma \left(2\theta +1\right)}+\frac{\theta^{3}t^{3\theta }}{\Gamma \left(3\theta +1\right)}\right]+\cdots \end{array}\;$$ (51)

It is worth noting that the solution at order θ=1 equals $e^{\mathrm{\alpha t}}\sin\left(x\right)$ , which corresponds to the exact solution of Equation (49).

Example 6.2. Consider the linear fractional S-H equation:

$${\;}^{\mathrm{AB}}D_{t}^{\theta }\varpi +\left(1-\alpha \right)\varpi +2\varpi_{x}+\varpi_{\text{xxxx}}=0,0<\theta \;\leq \;1,$$ (52)

with the condition:

ϖ(x,0)=cosx (53)

Solution. Based on the above method derivation, Equation (24) provides the foundation for determining the iterations, the solution proceeds as follows:

$$\varpi_{0}=\cos\left(x\right)\#\left(69\right)$$

$$\begin{array}{c} \varpi_{1}=\varpi \left(x,0\right)-H^{-1}\left[\left(\theta {\sqrt{A}}^{\theta }+1-\theta \right)H\left\{\left(1-\alpha \right)\varpi_{0}+2\varpi_{0_{x}}+\varpi_{0_{\text{xxxx}}}\right\}\right]\\ =\cos\left(x\right)+\alpha \cos\left(x\right)\left(\frac{\theta t^{\theta }}{\Gamma \left(\theta +1\right)}+1-\theta \right) \end{array}$$

$$\begin{array}{c} \varpi_{2}=\varpi \left(x,0\right)-H^{-1}\left[\left(\theta {\sqrt{A}}^{\theta }+1-\theta \right)H\left\{\left(1-\alpha \right)\varpi_{1}+2\varpi_{1_{x}}+\varpi_{1_{\text{xxxx}}}\right\}\right]\\ =\cos\left(x\right)+\alpha \cos\left(x\right)\left(1-\theta +\frac{\theta t^{\theta }}{\Gamma \left(\theta +1\right)}\right)+\alpha^{2}\cos\left(x\right)\left[{\left(1-\theta \right)}^{2}+2\left(1-\theta \right)\frac{\theta t^{\theta }}{\Gamma \left(\theta +1\right)}+\frac{\theta^{2}t^{2\theta }}{\Gamma \left(2\theta +1\right)}\right] \end{array}$$

$$\begin{array}{c} \varpi_{3}=\cos\left(x\right)+\alpha \cos\left(x\right)\left(1-\theta +\frac{\theta t^{\theta }}{\Gamma \left(\theta +1\right)}\right)+\alpha^{2}\cos\left(x\right)\left[{\left(1-\theta \right)}^{2}+2\left(1-\theta \right)\frac{\theta t^{\theta }}{\Gamma \left(\theta +1\right)}+\frac{\theta^{2}t^{2\theta }}{\Gamma \left(2\theta +1\right)}\right]\\ +\alpha^{3}\cos\left(x\right)\left[{\left(1-\theta \right)}^{3}+3{\left(1-\theta \right)}^{2}\frac{\theta t^{\theta }}{\Gamma \left(\theta +1\right)}+3\left(1-\theta \right)\theta^{2}\frac{t^{2\theta }}{\Gamma \left(2\theta +1\right)}+\frac{\theta^{3}t^{3\theta }}{\Gamma \left(3\theta +1\right)}\right] \end{array}$$

The solution can be written in series form:

$$\begin{array}{c} \varpi \left(x,t\right)=\cos\left(x\right)+\alpha \cos\left(x\right)\left(1-\theta +\frac{\theta t^{\theta }}{\Gamma \left(\theta +1\right)}\right)\\ +\alpha^{2}\cos\left(x\right)\left[{\left(1-\theta \right)}^{2}+2\left(1-\theta \right)\frac{\theta t^{\theta }}{\Gamma \left(\theta +1\right)}+\frac{\theta^{2}t^{2\theta }}{\Gamma \left(2\theta +1\right)}\right]\\ +\alpha^{3}\cos\left(x\right)\left[{\left(1-\theta \right)}^{3}+3{\left(1-\theta \right)}^{2}\frac{\theta t^{\theta }}{\Gamma \left(\theta +1\right)}+3\left(1-\theta \right)\theta^{2}\frac{t^{2\theta }}{\Gamma \left(2\theta +1\right)}+\frac{\theta^{3}t^{3\theta }}{\Gamma \left(3\theta +1\right)}\right]+\cdots \end{array}$$ (54)

It should be noted that the solution at order θ=1 equals $e^{\mathrm{\alpha t}}\cos\left(x\right)$ , which represents the exact solution of Equation (52).

Example 6.3. Consider the nonlinear fractional S-H equation:

$${\;}^{\mathrm{AB}}D_{t}^{\theta }\varpi +\left(1-\alpha \right)\varpi +2\varpi_{x}+\varpi_{\text{xxxx}}-\varpi^{2}+{\left(\varpi_{x}\right)}^{2}=0,\;0<\theta \;\leq \;1,$$ (55)

with the initial condition:

$$\varpi \left(x,0\right)=e^{x}.$$ (56)

Solution. Equation (24) can be applied to obtain

$$\varpi_{0}=e^{x}$$

$$\begin{array}{c} \varpi_{1}=\varpi \left(x,0\right)-H^{-1}\left[\left(\theta {\sqrt{A}}^{\theta }+1-\theta \right)H\left\{\left(1-\alpha \right)\varpi_{0}+2\varpi_{0_{x}}+\varpi_{0_{\text{xxxx}}}-\varpi_{0}^{2}+{\left(\varpi_{0_{x}}\right)}^{2}\right\}\right]\\ =e^{x}+\left(\alpha -4\right)e^{x}\left(1-\theta +\frac{\theta t^{\theta }}{\Gamma \left(\theta +1\right)}\right) \end{array}$$

$$\begin{array}{c} \varpi_{2}=\varpi \left(x,0\right)-H^{-1}\left[\left(\theta {\sqrt{A}}^{\theta }+1-\theta \right)H\left\{\left(1-\alpha \right)\varpi_{1}+2\varpi_{1_{x}}+\varpi_{1_{\text{xxxx}}}-\varpi_{1}^{2}+{\left(\varpi_{1_{x}}\right)}^{2}\right\}\right]\\ =e^{x}+\left(\alpha -4\right)e^{x}\left(1-\theta +\frac{\theta t^{\theta }}{\Gamma \left(\theta +1\right)}\right)+{\left(\alpha -4\right)}^{2}e^{x}\left[{\left(1-\theta \right)}^{2}+2\left(1-\theta \right)\frac{\theta t^{\theta }}{\Gamma \left(\theta +1\right)}+\frac{\theta^{2}t^{2\theta }}{\Gamma \left(2\theta +1\right)}\right] \end{array}$$

$$\begin{array}{c} \varpi_{3}=e^{x}+\left(\alpha -4\right)e^{x}\left(1-\theta +\frac{\theta t^{\theta }}{\Gamma \left(\theta +1\right)}\right)+{\left(\alpha -4\right)}^{2}e^{x}\left[{\left(1-\theta \right)}^{2}+2\left(1-\theta \right)\frac{\theta t^{\theta }}{\Gamma \left(\theta +1\right)}+\frac{\theta^{2}t^{2\theta }}{\Gamma \left(2\theta +1\right)}\right]\\ +{\left(\alpha -4\right)}^{3}e^{x}\left[{\left(1-\theta \right)}^{3}+3{\left(1-\theta \right)}^{2}\theta \frac{\theta t^{\theta }}{\Gamma \left(\theta +1\right)}+3\left(1-\theta \right)\theta^{2}\frac{t^{2\theta }}{\Gamma \left(2\theta +1\right)}+\frac{\theta^{3}t^{3\theta }}{\Gamma \left(3\theta +1\right)}\right]. \end{array}$$

The solution can be written in series form:

$$\begin{array}{c} \varpi \left(x,t\right)=e^{x}+\left(\alpha -4\right)e^{x}\left(1-\theta +\frac{\theta t^{\theta }}{\Gamma \left(\theta +1\right)}\right)+{\left(\alpha -4\right)}^{2}e^{x}\left[{\left(1-\theta \right)}^{2}+2\left(1-\theta \right)\frac{\theta t^{\theta }}{\Gamma \left(\theta +1\right)}+\frac{\theta^{2}t^{2\theta }}{\Gamma \left(2\theta +1\right)}\right]+\\ {\left(\alpha -4\right)}^{3}e^{x}\left[{\left(1-\theta \right)}^{3}+3{\left(1-\theta \right)}^{2}\theta \frac{\theta t^{\theta }}{\Gamma \left(\theta +1\right)}+3\left(1-\theta \right)\theta^{2}\frac{t^{2\theta }}{\Gamma \left(2\theta +1\right)}+\frac{\theta^{3}t^{3\theta }}{\Gamma \left(3\theta +1\right)}\right]+\cdots \end{array}$$ (57)

One should observe that the solution at θ=1 is equal to $e^{x+\left(\alpha -4\right)t}$ , which gives the exact solution of Equation (55).

**Figure 1.**
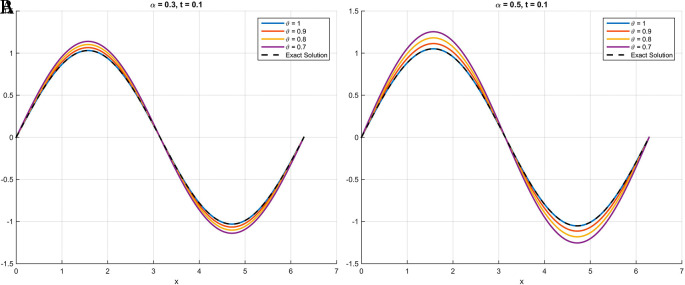
In Example 6.1, plots (A) and (B) illustrate that the curve increasingly converges toward the exact solution as θ approaches 1. Specifically, at θ = 0 *.*9, the curve nearly overlaps with that of θ = 1.

As illustrated in Figures 1 through 3, the numerical solutions obtained using the scheme based on the Atangana-Baleanu fractional derivative demonstrate a consistent convergence towards the exact analytical solution as the parameter θ approaches 1 . This behavior, where the curve at θ=0.9 nearly overlaps with that of the exact solution at θ=1 , validates the accuracy and efficacy of the proposed method. These qualitative findings are strongly reinforced by the absolute error tables ( Table 1 for Example 6.1, Table 2 for Example 6.2, and Table 3 for Example 6.3), which clearly demonstrate a significant decrease in error values as θ increases, providing compelling quantitative evidence for the robustness and precision of the proposed scheme.

**Figure 2.**
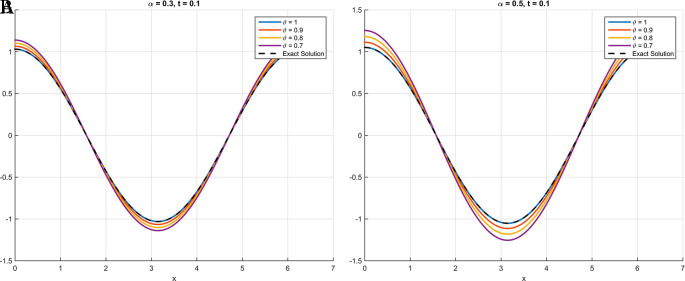
In Example 6.2, plots (A) and (B) illustrate that the curve increasingly converges toward the exact solution as *θ* approaches 1. Specifically, at *θ* = 0 *.*9, the curve nearly overlaps with that of *θ* = 1.

**Figure 3.**
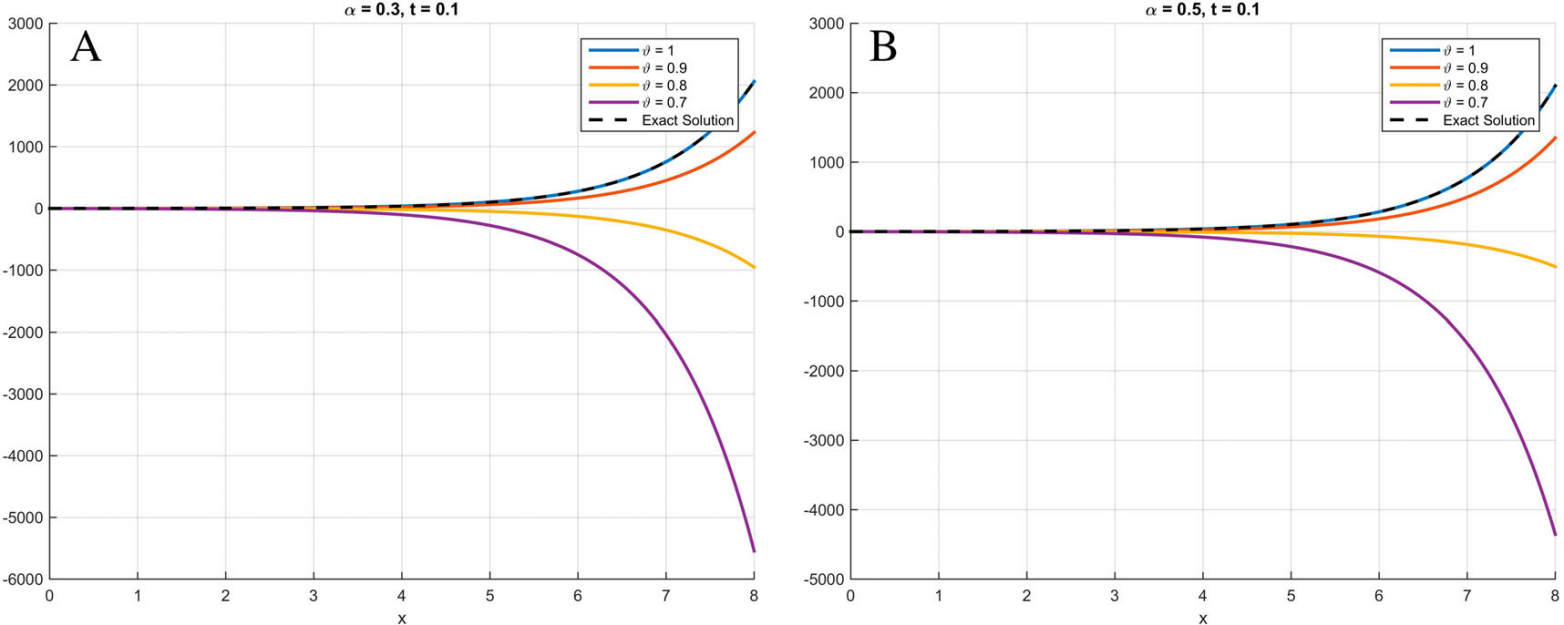
In Example 6.3, plots (A) and (B) illustrate that the curve increasingly converges toward the exact solution as *θ* approaches 1. Specifically, at *θ* = 0 *.*9, the curve nearly overlaps with that of *θ* = 1.

**Table 1.** A table showing the absolute error for Example 6.1, where the Atangana-Baleanu operator is used.

*t*	*x*	*AE*_*θ* = 1_	*AE*_*θ* = 0 *.*9_	*AE*_*θ* = 0 *.*8_	* AE*_*θ* = 0 *.*7_
0.1	0	0	0	0	0
	0.2	5.2259 × 10 *−*8	0.012341	0.025804	0.040299
	0.4	1.0243 × 10 *−*7	0.024191	0.050579	0.078991
	0.6	1.4853 × 10 *−*7	0.035076	0.073338	0.114530
	0.8	1.8870 × 10 *−*7	0.044562	0.093173	0.145510
	1	2.2134 × 10 *−*7	0.052272	0.109290	0.170690
0.5	0	0	0	0	0
	0.2	3.4022 × 10 *−*5	0.011583	0.022722	0.033049
	0.4	6.6688 × 10 *−*5	0.022705	0.044538	0.064780
	0.6	9.6695 × 10 *−*5	0.032921	0.064579	0.093929
	0.8	0.000122850	0.041825	0.082045	0.119330
	1	0.000144100	0.049061	0.096240	0.139980

**Table 2.** A table showing the absolute error for Example 6.2, where the Atangana-Baleanu operator is used.

*t*	x	*AE*_*θ* = 1_	*AE*_*θ* = 0 *.*9_	*AE*_*θ* = 0 *.*8_	* AE*_*θ* = 0 *.*7_
0.1	0	2.6304 × 10 *−*7	0.062120	0.129880	0.20284
	0.2	2.5780 × 10 *−*7	0.060882	0.127290	0.19880
	0.4	2.4228 × 10 *−*7	0.057216	0.119630	0.18683
	0.6	2.1710 × 10 *−*7	0.051270	0.107200	0.16741
	0.8	1.8326 × 10 *−*7	0.043279	0.090491	0.14132
	1	1.4212 × 10 *−*7	0.033564	0.070177	0.10960
0.5	0	0.00017125	0.058304	0.114370	0.16635
	0.2	0.00016784	0.057142	0.112090	0.16304
	0.4	0.00015773	0.053702	0.105340	0.15322
	0.6	0.00014134	0.048121	0.094395	0.13730
	0.8	0.00011931	0.040621	0.079683	0.11590
	1	9.2527 × 10 *−*5	0.031502	0.061795	0.08988

**Table 3.** A table showing the absolute error for Example 6.3, where the Atangana-Baleanu operator is used.

*t*	*x*	*AE*_*θ* = 1_	*AE*_*θ* = 0 *.*9_	*AE*_*θ* = 0 *.*8_	* AE*_*θ* = 0 *.*7_
0.1	-6	1.4474 × 10 *−*6	0.00062413	0.0021663	0.0053680
	-5.8	1.7679 × 10 *−*6	0.00076231	0.0026459	0.0065565
	-5.6	2.1593 × 10 *−*6	0.00093109	0.0032317	0.0080082
	-5.4	2.6373 × 10 *−*6	0.00093109	0.0039472	0.0097812
	-5.2	3.2212 × 10 *−*6	0.00138900	0.0048211	0.0119470
	-5	3.9344 × 10 *−*6	0.00169660	0.0058885	0.0145920
0.5	-6	0.00070831	0.0047265	0.012274	0.022038
	-5.8	0.00086513	0.0057730	0.014991	0.026917
	-5.6	0.00105670	0.0070512	0.018311	0.032877
	-5.4	0.00129060	0.0086123	0.022365	0.040156
	-5.2	0.00157640	0.0105190	0.027316	0.049047
	-5	0.00192540	0.0128480	0.033364	0.059906

## 7. Conclusion

This work tackles the Swift–Hohenberg equation, a pivotal partial differential equation in engineering and physics. Our contribution is a novel semi-analytical technique founded on a fusion of the variational iteration method and the newly introduced Yasser–Jassim transform. The proposed method’s convergence is rigorously examined, and sufficient conditions are formulated to ensure reliable results.

The adoption of the Atangana–Baleanu fractional derivative further provides a more accurate framework for solution description and enhances the model’s adaptability to practical scenarios. Numerical experiments, supported by graphical illustrations and absolute error tables, validated the efficiency of the proposed methodology. The stability of the solution under the fractional derivative framework was also rigorously investigated and proven. Notably, the results demonstrate that even the third iterative solution—showcased in the absolute error tables—delivers the accuracy required for real-world implementation.

The model’s capacity for effortless derivation of higher-order iterations offers a clear pathway to achieving even greater precision and robustness. Overall, the findings confirm that the proposed approach is effective, robust, and extendable, making it a powerful tool not only for solving fractional-order partial differential equations but also for tackling ordinary and integral equations in future applications.

## Ethics statement

This research does not involve human participants, animal subjects, or sensitive personal data. Therefore, ethical approval was not required.

## Data Availability

All data generated or analysed during this study are included in this published article. No additional datasets were used or created.
