# Supplementary material for: A New Analytical Method for Solving the Fractional Swift-Hohenberg Equation with Atangana-Baleanu Derivative
Source: F1000Res. 2025 Dec 16;14:1402. [Version 1] doi: 10.12688/f1000research.172948.1 (PMC13182787; doi:10.12688/f1000research.172948.1)
Supplement: Peer review history

# Reviewer report: Reviewer response for version 1

**DOI:** 10.5256/f1000research.190718.r463626

**Published:** 2026-03-25

**Authors:** Mountassir Hamdi Cherif

**Affiliations:** 1 Hight School of Electrical and Energetics Engineering of Oran (ESGEE-Oran), Oran, Algeria

**1. Summary of the Research**

 The manuscript addresses the fractional-order Swift-Hohenberg (S-H) equation, a fundamental model in pattern formation and fluid dynamics. The authors propose a novel hybrid analytical approach by combining the **Yasser-Jassim Integral Transform** with the **Variational Iteration Method (VIM)**.

 To enhance the physical modeling of memory effects, the authors employ the **Atangana-Baleanu (AB) fractional derivative** in the Caputo sense. The study includes a rigorous theoretical framework, providing proofs for the existence and uniqueness of the solution using fixed-point theorems, as well as a detailed convergence analysis. The method is then validated through numerical examples, demonstrating its efficiency in producing series solutions that converge rapidly to the exact solution as the fractional order approaches unity.

**2. General Remarks**

- **Originality:** The integration of the Yasser-Jassim transform into the VIM framework for the AB derivative is a noteworthy contribution. It simplifies the determination of Lagrange multipliers for non-local operators.
- **Clarity:** The mathematical derivations are presented logically, and the transition from the integral transform to the iterative scheme is well-documented.
- **Significance:** The Swift-Hohenberg equation is a complex fourth-order equation; finding efficient semi-analytical solutions for its fractional counterpart is highly relevant for researchers in mathematical physics and nonlinear dynamics.

**3. Specific Remarks and Technical Errors**

 While the paper is technically sound, the following points should be addressed to improve the quality of the final index:

- **Notation Consistency:** In the section defining the Atangana-Baleanu derivative, ensure that the normalization function B(\theta) is explicitly defined or referenced as satisfying B(0) = B(1) = 1.
- **Numerical Comparison:** In Section 6, while the convergence to the exact solution is shown, the authors should ideally provide a table of absolute errors for different values of t and x. This would quantitatively demonstrate the precision of the YJVIM.
- **Typographical Errors:**
  - Check for consistent spacing in the multi-line equations (e.g., Equation 30 and 31) to ensure symbols do not overlap with the margins.
  - Verify the subscripts in the recurrence relations to ensure they align correctly with the iteration index n.
- **Literature Review:** While the authors cite recent works on the Yasser-Jassim transform, adding a brief mention of how this specific hybrid method differs in computational complexity from the Laplace-Variational Iteration Method (LVIM) would provide better context for the reader.

**4. Conclusion and Recommendation**

**Recommendation: Accept with Minor Revisions.**

 The manuscript presents a robust and well-validated analytical framework for solving a challenging class of fractional differential equations. The theoretical proofs are sound, and the application to the Swift-Hohenberg equation is successful. Once the minor typographical corrections and the suggested clarifications in the numerical section are addressed, the paper will be a valuable addition to the journal.

Is the work clearly and accurately presented and does it cite the current literature?

Yes

If applicable, is the statistical analysis and its interpretation appropriate?

Not applicable

Are all the source data underlying the results available to ensure full reproducibility?

Partly

Is the study design appropriate and is the work technically sound?

Yes

Are the conclusions drawn adequately supported by the results?

Yes

Are sufficient details of methods and analysis provided to allow replication by others?

Yes

Reviewer Expertise:

Fractional derivatives and integrals, Analytical methods to solve fractional and local fractional ODEs and PDEs, Integral Transforms.

I confirm that I have read this submission and believe that I have an appropriate level of expertise to confirm that it is of an acceptable scientific standard.

# Reviewer report: Reviewer response for version 1

**DOI:** 10.5256/f1000research.190718.r460718

**Published:** 2026-03-11

**Authors:** Nguyen Minh Tuan

**Affiliations:** 1 Posts and Telecommunications Institute of Technology, Ho Chi Minh City, Vietnam

Review: A New Analytical Method for Solving the Fractional Swift-Hohenberg Equation with Atangana-Baleanu Derivative:

 This work introduces a hybrid analytical technique for solving the Swift–Hohenberg equation by integrating the newly formulated Yasser–Jassim integral transform with the variational iteration method.

1. Are Eq. (2), (3), (4) new or got from anywhere? It should be clear. As I can see, it is same at the paper namely Fractional View Analysis of Swift–Hohenberg Equations by an Analytical Method and Some Physical Applications by Salemah A. Almutlak, Rasool Shah, Wajaree Weera, Samir A. El-Tantawy and Lamiaa S. El-Sherif.
2. What do Theorems 2.1 and 2.2 use for? I think it belongs to another problem.
3. In Section 3, Eq. 24 is the result of integral transformation then could attain the weak solution. Does it derive an analytic or strong solution? Should follow Gronwall-Bellman inequality.
4. Does Section 4 need to be performed? Followed Theorem Arzela-Ascoli Theorem, it seems redundant.
5. Section 5 needs to be clarified. What role does it play in the paper? Is it necessary?
6. From Definition 2.4, theta is in (0,1) but in example 6.1-6.3, theta has gotten equally 1. Should it be clarified?
7. Exact solutions are not satisfied with the original equation. Should it be clarified?
8. English grammar is confused and seems using AI. I don’t have AI checking applications. Please check it again, especially in introduction and conclusion. We should give statement about this.
9. Some properties are not related to the content, for example, at page 4. Should we omit them?

Is the work clearly and accurately presented and does it cite the current literature?

Yes

If applicable, is the statistical analysis and its interpretation appropriate?

No

Are all the source data underlying the results available to ensure full reproducibility?

No

Is the study design appropriate and is the work technically sound?

Yes

Are the conclusions drawn adequately supported by the results?

No

Are sufficient details of methods and analysis provided to allow replication by others?

Yes

Reviewer Expertise:

Applied mathematics and computer science

I confirm that I have read this submission and believe that I have an appropriate level of expertise to confirm that it is of an acceptable scientific standard, however I have significant reservations, as outlined above.

# Response

**Published:** 2026-03-13

**Authors:** Mohammed Taimah

**Affiliations:** Not Applicable, Iraq

We sincerely thank the reviewer for the valuable comments and constructive suggestions.

**Response 1:**

 Equations (2), (3), and (4) are not new and are not introduced as original contributions of the present work. They are well-known formulations of the Swift–Hohenberg equation and have appeared in several previous studies, including the paper mentioned by the reviewer. This has been clarified explicitly in the revised manuscript.

**Response 2:**

 Theorems 2.1 and 2.2 are used in the section **“Stability Analysis of YJVIM”** and are essential for establishing the theoretical basis of the stability discussion presented in the manuscript.

**Response 3:**

 In this work, the analytical exact solution of the fractional-order equation is obtained. Therefore, Equation (24) is not intended merely as a weak solution resulting from the integral transformation, but rather as part of the derivation of the exact analytical solution of the considered fractional problem.

**Response 4:**

 Section 4 is necessary because it provides the stability analysis of the obtained solutions and strengthens the theoretical framework of the manuscript. Therefore, it has been retained as an essential part of the study.

**Response 5:**

 Section 5 is also necessary, as it plays an important role in ensuring the convergence of the obtained solutions when applying the proposed method. For this reason, it has been retained and clarified in the revised manuscript.

**Response 6:**

 This point has been clarified in the revised manuscript. Although the fractional order (\theta) is defined in ((0,1)), it is common in similar studies to present, at the end of the examples, the corresponding classical exact solution when the order becomes (1). In our work, the tables clearly include different fractional values such as (0.9), (0.7), and (1), where (\theta = 1) is used only to represent the classical integer-order case for comparison purposes.

**Response 7:**

 This point has been clarified in the revised manuscript. The exact solution of the fractional-order equation is generally expressed in the form of an infinite series, whereas the exact solution presented in the examples corresponds to the classical case when the order is (1). This comparison is commonly adopted in related studies to illustrate the consistency of the obtained results with the classical model.

**Response 8:**

 The manuscript has been carefully revised again, and the English language has been improved as much as possible throughout the paper, particularly in the Introduction and Conclusion sections, in order to enhance clarity and readability.

**Response 9:**

 This comment has been addressed in the revised manuscript. Two properties that were not sufficiently related to the main content have been removed, while the two remaining properties were retained because they are directly relevant to the present study.

# Reviewer report: Reviewer response for version 1

**DOI:** 10.5256/f1000research.190718.r463627

**Published:** 2026-03-09

**Authors:** Enes Ata

**Affiliations:** 1 Bingol University, Bingol, Turkey

The manuscript has the potential to provide scientific novelty and may offer guidance to young researchers in this field. However, minor revisions are required before it can be accepted for indexing. These are listed below:

 1.    In the introduction section, the authors should discuss recent scientific studies on fractional calculus and include them in the references. These studies are listed below:

 •    Reference 1

 •    Reference 2

 •    Reference 3

 •    Reference 4

 •    Reference 5

 •    Reference 6

 •    Reference 7

 2. Appropriate spacing should be inserted between consecutively presented convergence conditions in the mathematical expressions.

 3. Mathematical formulas given consecutively should be properly aligned.

 4. Since the normalization function is already mentioned in Equation (7), there is no need to restate it in Equation (8); please remove it.

 5. The English language of the manuscript should be polished.

 6. Please label the vertical axes of the provided graphs.

 7. Verify the data presented in the tables.

 8. In the conclusion section, please include statements focusing on possible directions for future work.

 Note: The authors are kindly requested to address the above revisions thoroughly. I would like to thank them for their efforts and wish them success in their future studies.

Is the work clearly and accurately presented and does it cite the current literature?

Partly

If applicable, is the statistical analysis and its interpretation appropriate?

Partly

Are all the source data underlying the results available to ensure full reproducibility?

Partly

Is the study design appropriate and is the work technically sound?

Partly

Are the conclusions drawn adequately supported by the results?

Partly

Are sufficient details of methods and analysis provided to allow replication by others?

Partly

Reviewer Expertise:

Fractional calculus; integral transforms; special functions; differential equtions; mathematical modeling, electrical circuits; heat transfer. etc

I confirm that I have read this submission and believe that I have an appropriate level of expertise to confirm that it is of an acceptable scientific standard, however I have significant reservations, as outlined above.

## References

[1] : A new collection of real world applications of fractional calculus in science and engineering. *Communications in Nonlinear Science and Numerical Simulation* .2018;64: 10.1016/j.cnsns.2018.04.019 213-231 10.1016/j.cnsns.2018.04.019

[2] : New generalized Mellin transform and applications to partial and fractional differential equations. *International Journal of Mathematics and Computer in Engineering* .2023;1(1) : 10.2478/ijmce-2023-0004 45-66 10.2478/ijmce-2023-0004

[3] : A new study on the mathematical modelling of human liver with Caputo–Fabrizio fractional derivative. *Chaos, Solitons & Fractals* .2020;134: 10.1016/j.chaos.2020.109705 10.1016/j.chaos.2020.109705

[4] : Special functions with general kernel: Properties and applications to fractional partial differential equations. *International Journal of Mathematics and Computer in Engineering* .2025;3(2) : 10.2478/ijmce-2025-0013 153-170 10.2478/ijmce-2025-0013

[5] : Stability analysis of Caputo fractional-order nonlinear systems revisited. *Nonlinear Dynamics* .2012;67(4) : 10.1007/s11071-011-0157-5 2433-2439 10.1007/s11071-011-0157-5

[6] : Generalized Gamma, Beta and Hypergeometric Functions Defined by Wright Function and Applications to Fractional Differential Equations. *Cumhuriyet Science Journal* .2022;43(4) : 10.17776/csj.1005486 684-695 10.17776/csj.1005486

[7] : A new fractional model and optimal control of a tumor-immune surveillance with non-singular derivative operator. *Chaos: An Interdisciplinary Journal of Nonlinear Science* .2019;29(8) : 10.1063/1.5096159 10.1063/1.5096159 31472488

# Response

**Published:** 2026-03-13

**Authors:** Mohammed Taimah

**Affiliations:** Not Applicable, Iraq

We sincerely thank the reviewer for the valuable comments and constructive suggestions.

**Response 1:**

 The suggested references were carefully reviewed, and the most relevant studies related to the general development of fractional calculus were selected. Accordingly, References **1, 3, and 7** have been incorporated into the revised introduction section.

**Response 2:**

 The spacing between consecutively presented convergence conditions has been corrected throughout the manuscript.

**Response 3:**

 The consecutively presented mathematical formulas have been revised and aligned as much as possible in the updated manuscript. Any minor remaining inconsistencies are due to the formatting limitations of the journal template.

**Response 4:**

 The repeated normalization function has been removed from Equation (8).

**Response 5:**

 The manuscript has been thoroughly proofread, and the English language has been improved throughout the paper.

**Response 6:**

 The figures were carefully checked again. Since the plotted quantities are already clear from the context and captions, the current graphical presentation was retained in order to maintain visual clarity and consistency.

**Response 7:**

 The data presented in the tables have been carefully rechecked and verified.

**Response 8:**

 The conclusion section has been revised to include a brief statement on possible future research directions.
